# The Escherichia coli β-Barrel Assembly Machinery Is Sensitized to Perturbations under High Membrane Fluidity

**DOI:** 10.1128/JB.00517-18

**Published:** 2018-12-07

**Authors:** Kelly M. Storek, Rajesh Vij, Dawei Sun, Peter A. Smith, James T. Koerber, Steven T. Rutherford

**Affiliations:** a Department of Infectious Diseases, Genentech, Inc., South San Francisco, California, USA; b Department of Antibody Engineering, Genentech, Inc., South San Francisco, California, USA; c Department of Structural Biology, Genentech, Inc., South San Francisco, California, USA; Michigan State University

**Keywords:** BamA, BamB, OMPs, membrane fluidity, outer membrane

## Abstract

Integral β-barrel membrane proteins are folded and inserted into the Gram-negative bacterial outer membrane by the β-barrel assembly machine (BAM). This essential complex, composed of a β-barrel protein, BamA, and four lipoproteins, BamB, BamC, BamD, and BamE, resides in the outer membrane, a unique asymmetrical lipid bilayer that is difficult to recapitulate *in vitro*. Thus, the probing of BAM function in living cells is critical to fully understand the mechanism of β-barrel folding. We recently identified an anti-BamA monoclonal antibody, MAB1, that is a specific and potent inhibitor of BamA function. Here, we show that the inhibitory effect of MAB1 is enhanced when BAM function is perturbed by either lowering the level of BamA or removing the nonessential BAM lipoproteins, BamB, BamC, or BamE. The disruption of BAM reduces BamA activity, increases outer membrane (OM) fluidity, and activates the σ^E^ stress response, suggesting the OM environment and BAM function are interconnected. Consistent with this idea, an increase in the membrane fluidity through changes in the growth environment or alterations to the lipopolysaccharide in the outer membrane is sufficient to provide resistance to MAB1 and enable the BAM to tolerate these perturbations. Amino acid substitutions in BamA at positions in the outer membrane spanning region or the periplasmic space remote from the extracellular MAB1 binding site also provide resistance to the inhibitory antibody. Our data highlight that the outer membrane environment is a critical determinant in the efficient and productive folding of β-barrel membrane proteins by BamA.

**IMPORTANCE** BamA is an essential component of the β-barrel assembly machine (BAM) in the outer membranes of Gram-negative bacteria. We have used a recently described inhibitory anti-BamA antibody, MAB1, to identify the molecular requirements for BAM function. Resistance to this antibody can be achieved through changes to the outer membrane or by amino acid substitutions in BamA that allosterically affect the response to MAB1. Sensitivity to MAB1 is achieved by perturbing BAM function. By using MAB1 activity and functional assays as proxies for BAM function, we link outer membrane fluidity to BamA activity, demonstrating that an increase in membrane fluidity sensitizes the cells to BAM perturbations. Thus, the search for potential inhibitors of BamA function must consider the membrane environment in which β-barrel folding occurs.

## Abstract (precis)

BamA is an essential component of the β-barrel assembly machine (BAM) in the outer membranes of Gram-negative bacteria. We have used a recently described inhibitory anti-BamA antibody, MAB1, to identify the molecular requirements for BAM function. Resistance to this antibody can be achieved through changes to the outer membrane or by amino acid substitutions in BamA that allosterically affect the response to MAB1. Sensitivity to MAB1 is achieved by perturbing BAM function. By using MAB1 activity and functional assays as proxies for BAM function, we link outer membrane fluidity to BamA activity, demonstrating that an increase in membrane fluidity sensitizes the cells to BAM perturbations. Thus, the search for potential inhibitors of BamA function must consider the membrane environment in which β-barrel folding occurs.

## INTRODUCTION

The outer membranes (OMs) of Gram-negative bacteria are permeability barriers to cytotoxic molecules such as detergents and antibiotics (1). The lipids comprising the OM are asymmetrically organized, with phospholipids occupying the inner leaflet and lipopolysaccharide (LPS) confined to the outer leaflet (2, 3). Rigidity and impermeability are imparted to the OM by tight lateral interactions between adjacent LPS molecules mediated by divalent cations and dense packing of LPS and phospholipid hydrocarbon chains (1). A disruption of this permeability barrier results in an increased sensitivity to antibiotics that are typically excluded.

Proteins embedded within the OM perform critical cellular processes, including nutrient acquisition, toxin efflux, and LPS transport (4). Integral OM proteins (OMPs) typically assume a β-barrel fold in which an amphipathic β-sheet is wrapped such that the first and last β-strands are adjacent (5). These β-barrel OMPs are synthesized in the cytoplasm, secreted into the aqueous periplasm by the Sec machinery, and interact with chaperones before ultimately being folded and inserted into the OM. The process of folding and inserting OMPs in Gram-negative bacteria is essential for their viability and requires the dedicated β-barrel assembly machine (BAM) (6, 7).

BAM is a multiprotein OM complex. BamA, the central component of the BAM complex, is composed of a β-barrel OMP and five periplasmic polypeptide transport-associated (POTRA) domains (8–10). Four OM lipoproteins, BamB, BamC, BamD, and BamE, interact with the BamA POTRA domains (9–11). Only BamA and BamD are essential for viability (4, 6, 12, 13); however, all five components are needed for maximal β-barrel folding activity in a reconstituted *in vitro* system (14, 15). Moreover, Escherichia coli cannot tolerate the simultaneous loss of BamC and BamE or of BamB and BamE (6, 12, 13, 16).

There are multiple models to described β-barrel folding by BAM and the functionally related sorting and assembly machine (SAM) found in eukaryotes; however, the precise molecular mechanism remains unknown (9, 10, 17–25). Only recently have specific and potent modulators of β-barrel folding become available to tease apart individual steps in this process (26, 27). Previously, we described an anti-BamA monoclonal antibody, MAB1, which inhibits OMP folding activity by binding directly to an extracellular loop of BamA in a strain with truncated LPS (27). E. coli is sensitized to the inhibitory effect of MAB1 when membrane fluidity is high, suggesting that BAM activity is sensitive to the state of the membrane environment in which it is embedded. Here, we explore this hypothesis by defining the molecular requirements for MAB1 activity. We identify BamA amino acid substitutions in the transmembrane and periplasmic domains that lead to MAB1 resistance and find that lowering BamA levels or removing nonessential BAM lipoproteins increases membrane fluidity and sensitizes E. coli to MAB1 inhibition. Our results suggest that optimal BAM activity is dependent on the bacterial membrane environment.

## RESULTS

### BAM activity is defective in E. coli Δ*waaD*.

The bactericidal anti-BamA monoclonal antibody MAB1 inhibits BamA function in the LPS-truncated E. coli Δ*waaD* strain (27). In addition to increasing the access to surface epitopes on BamA, the truncated LPS in the E. coli Δ*waaD* strain also increases OM fluidity without altering the BamA level (27–31). This excessively fluid membrane environment sensitizes the cells to inhibition by MAB1 (27), suggesting BAM function and membrane fluidity are linked. Consistent with this, σ^E^ activity, which responds to the accumulation of unfolded OMPs, is also elevated in E. coli Δ*waaD* (27, 32). We hypothesized that high membrane fluidity may cause a defect in BamA OMP folding activity, BAM complex assembly, or both.

**FIG 1:**
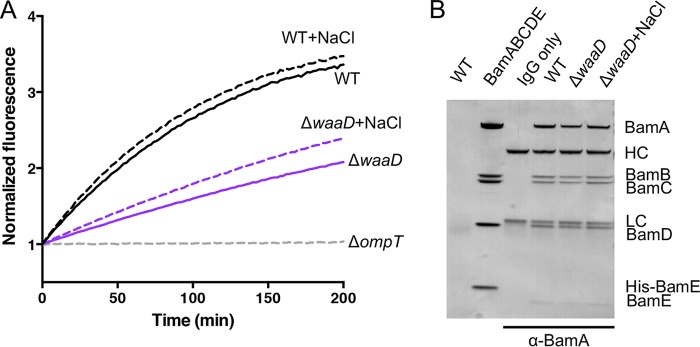
BAM activity is reduced in E. coli Δ*waaD*. (A) OmpT cleavage assay of bacterial strains grown in medium alone (solid lines) or in medium supplemented with 150 mM NaCl (dashed lines) for successful folding and insertion of OmpT by monitoring the increase in fluorescence upon substrate cleavage. A mutant strain lacking *ompT* served as a control. Experiments were performed in biological triplicates, and the composite curves are shown. (B) BAM complexes examined by co-IP using an anti-BamA antibody (MAB3). First lane (WT), no anti-BamA antibody control; second lane (BamABCDE), purified complex protein; third lane (IgG only), anti-BamA antibody; fourth through sixth lanes, co-IPs with parent (WT) and Δ*waaD* strains and the Δ*waaD* strain in medium with NaCl. BamA (91 kDa), BamB (42 kDa), BamC (37 kDa), BamD (28 kDa), His-BamE (13.4 kDa), BamE (12.3 kDa), antibody heavy chain (HC; ∼50 kDa), and antibody light chain (LC; ∼25 kDa) are indicated. An image with enhanced contrast showing BamE can be found in Fig. S1B in the supplemental material.

To monitor BAM activity in the E. coli Δ*waaD* strain, we used an OmpT protease assay as a proxy (14, 33, 34). OmpT is a BAM substrate and, upon proper folding and insertion into the OM, OmpT cleaves a self-quenching fluorogenic reporter peptide. The OmpT folding activity of BAM is determined by monitoring the increase in fluorescence over time. OmpT activity was lower in the E. coli Δ*waaD* strain than in the wild-type E. coli BW25113 (Fig. 1A). Upon growth in the presence of NaCl, which decreases membrane fluidity (27, 35), OmpT activity was increased (Fig. 1A). The total OMP profiles of wild-type E. coli BW25113 and E. coli Δ*waaD* were similar overall, with a slight trend toward fewer OMPs detected in the E. coli Δ*waaD* strain, indicating that although less efficient, BAM in E. coli Δ*waaD* was able to ultimately fold and insert the OMPs required for growth (see Fig. S1A in the supplemental material). Overall, these data were consistent with a connection between BAM activity and membrane fluidity.

To measure the formation of the multiprotein BAM complex, we performed BamA coimmunoprecipitation (co-IP) experiments using an anti-BamA monoclonal antibody with wild-type E. coli BW25113 and E. coli Δ*waaD* cells grown under high and low membrane fluidity conditions. The anti-BamA antibody pulled down equal amounts of BamA, BamB, BamC, and BamD for both strains in high and low NaCl (Fig. 1B). A band corresponding to BamE was detected at much lower levels than the other BAM lipoproteins but was unchanged across the samples (Fig. S1B). Thus, strains with different sensitivities to MAB1, membrane fluidities, and BamA activities formed BAM complexes at similar levels.

### E. coli Δ*waaD* cannot tolerate a decreased BamA level under high membrane fluidity conditions.

**FIG 2:**
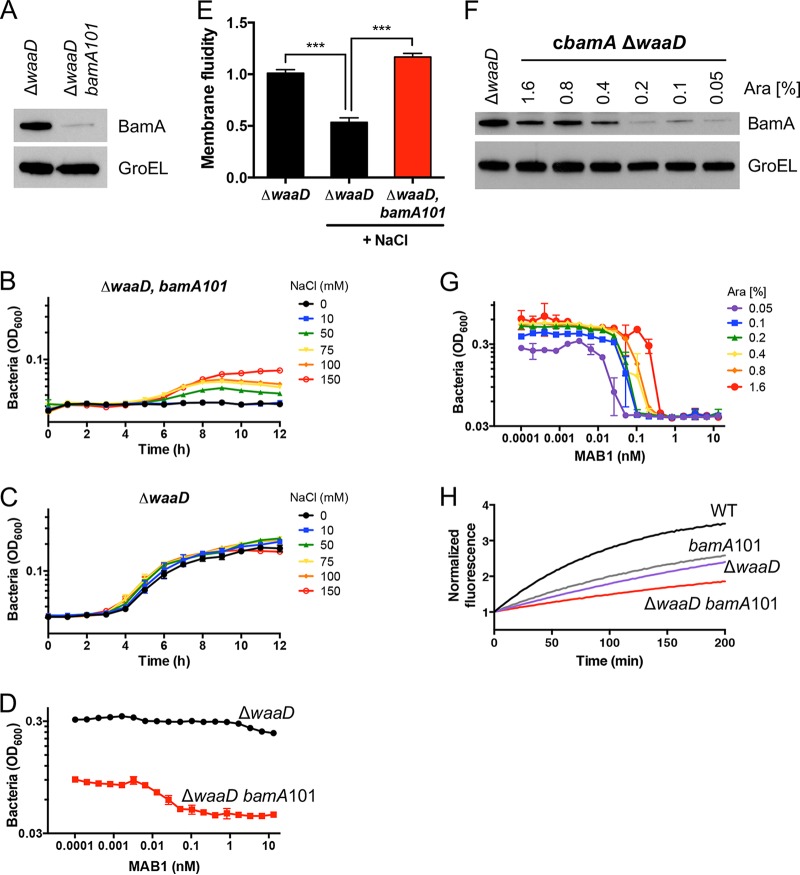
Low BamA levels affect membrane properties and increase MAB1 sensitivity. (A) Western blots of BamA and GroEL protein levels were compared from mid-log cultures grown in medium supplemented with 150 mM NaCl. (B and C) Bacterial growth curves for E. coli Δ*waaD bamA101* (B) and E. coli Δ*waaD* (C) grown with different concentrations of NaCl. (D) Bacterial growth of E. coli Δ*waaD bamA101* was inhibited in the presence of MAB1 after 6 h in medium supplemented with 150 mM NaCl. (E) Membrane fluidity was measured for each described strain in medium alone or medium supplemented with 150 mM NaCl. Data are expressed relative to E. coli Δ*waaD* grown in medium alone without added NaCl. (F) Western blots of BamA and GroEL protein levels from mid-log cultures. (G) Bacterial growth of an arabinose-inducible *bamA* E. coli Δ*waaD* strain measured after 20 h in the presence of MAB1 in medium supplemented with different concentrations of arabinose. (H) OmpT substrate cleavage assay for strains grown in medium supplemented with 150 mM NaCl. For all plotted experiments, means and standard deviations (SDs) from biological triplicates are shown. ***, *P < *0.001.

On the basis of the hypothesis that membrane fluidity and BAM activity are linked, we predicted that E. coli Δ*waaD* would be sensitized to low BamA levels when grown under conditions that promote high membrane fluidity. To test this, we constructed an E. coli Δ*waaD bamA101* double mutant. The Δ*waaD* deletion increases membrane fluidity (27) and compromises BamA function (Fig. 1A), and *bamA101* is a transposon insertion in the *bamA* promoter that reduces BamA levels by >90% compared to that of the E. coli Δ*waaD* parent (Fig. 2A) (36). This E. coli Δ*waaD bamA101* strain was maintained on high-NaCl medium, which lowers membrane fluidity (35); however, when it was moved to low-NaCl medium, which can affect multiple cellular processes, including increasing the membrane fluidity (35), the E. coli Δ*waaD bamA101* did not grow (see Fig. S2A and B). The addition of NaCl to the medium improved E. coli Δ*waaD bamA101* growth in a dose-dependent manner, but this did not affect the growth of the E. coli Δ*waaD* parent (Fig. 2B and C). In addition to adding NaCl, E. coli Δ*waaD bamA101* growth in low-NaCl medium was rescued by incubating at a low temperature, 30°C (Fig. S2C), another condition that, among other effects, reduces membrane fluidity (35, 37). Finally, the E. coli *bamA101* parent strain, which has LPS with a core oligosaccharide that has been shown to decrease OM fluidity (31, 38), exhibited wild-type growth (Fig. S2A and B). Thus, under high membrane fluidity conditions, E. coli Δ*waaD* cannot tolerate reduced BamA protein levels, and this was rescued by increasing the NaCl concentration, decreasing the growth temperature, or adding sugars to LPS, all of which have the common effect of decreasing membrane fluidity.

We predicted that the E. coli Δ*waaD bamA101* strain would also be sensitized to the inhibitory activity of MAB1, because it produced fewer BamA targets than the E. coli Δ*waaD* parent strain (Fig. 2A). MAB1 only inhibits the growth of E. coli Δ*waaD* in low NaCl, and so we were unable to compare the activity with that of the E. coli Δ*waaD bamA101* strain because of its inability to grow under these conditions (Fig. 2B). However, when grown under permissible high-NaCl conditions, E. coli Δ*waaD bamA101* was sensitized to MAB1 (Fig. 2D). This sensitization might have been due to the decreased target level caused by the *bamA101* allele, a secondary effect of this mutation on the OM (39), or a combination of both.

The state of the OM in the E. coli Δ*waaD bamA101* strain was assessed to determine the impact of the low BamA level. Membrane fluidity was measured by monitoring the lateral mobility of a membrane-embedded fluorescent probe (27, 37). E. coli Δ*waaD bamA101* exhibited significantly increased membrane fluidity relative to that of the E. coli Δ*waaD* parent strain (Fig. 2E). An ethidium bromide (EtBr) accumulation assay was used to monitor the effects on OM permeability (40). Consistent with lower BamA levels increasing the membrane permeability (39), E. coli Δ*waaD bamA101* exhibited increased EtBr accumulation compared to that of the parent E. coli Δ*waaD* strain (Fig. S2D). MAB1 sensitization by lower BamA levels was confirmed by constructing a conditional *bamA* mutant in which *bamA* was expressed from an arabinose-titratable promoter in an E. coli Δ*waaD* background (c*bamA* Δ*waaD*). In this strain, MAB1 activity decreased with increasing arabinose concentrations and BamA levels (Fig. 2F and G). The dosing of arabinose in a concentration range that supports bacterial growth from a low concentration (0.05%) to a higher concentration (1.6%) increased the MAB1 MIC 8-fold (Fig. 2G). Thus, the increased sensitivity of E. coli Δ*waaD bamA101* to MAB1 correlates with both BamA target level and membrane fluidity.

The OmpT reporter assay was used to test for effects of these mutants on intrinsic BAM activity. OmpT activity was decreased in both the E. coli Δ*waaD* strain and the E. coli *bamA101* strain, and the E. coli Δ*waaD bamA101* double mutant exhibited an even greater defect (Fig. 2H). This is consistent with our hypothesis that the intrinsic activity of BamA is defective in highly fluid membranes.

### BAM complexes lacking BamC or BamE are hypersensitive to MAB1.

**FIG 3:**
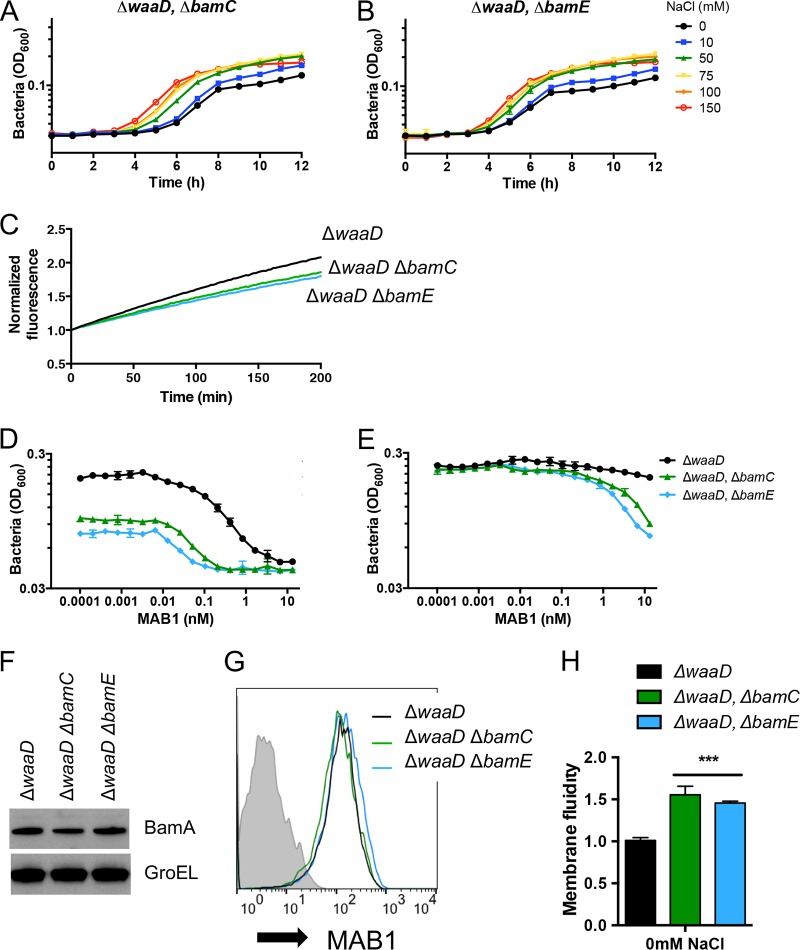
BAM complexes lacking BamC or BamE are hypersensitive to MAB1. (A and B) Bacterial growth curves for E. coli Δ*waaD* Δ*bamC* (A) and E. coli Δ*waaD* Δ*bamE* (B) with increasing concentrations of supplemented NaCl shown. (C) OmpT substrate cleavage assay for strains grown in medium alone. (D and E) Bacterial growth inhibition by MAB1 was measured by bacterial cell density (OD_600_) in medium alone (D) or medium supplemented with 150 mM NaCl (E). (F and G) BamA protein levels were compared from mid-log cultures by Western blot (F) and FACS (G). Representative FACS traces are shown. (H) Membrane fluidity was increased in E. coli Δ*waaD* Δ*bamC* and E. coli Δ*waaD* Δ*bamE*. Data are expressed relative to E. coli Δ*waaD* grown in medium alone. For all plotted experiments, means and SDs from biological triplicates are shown. ***, *P < *0.001.

BamA folding activity is optimal when all five members of the BAM complex are present (6, 12–14). To test the contributions of nonessential BAM lipoproteins to E. coli Δ*waaD* growth, we constructed E. coli Δ*waaD* Δ*bamC* and E. coli Δ*waaD* Δ*bamE* double mutants, which exhibited only slight growth defects under low-NaCl high membrane fluidity conditions (Fig. 3A and B). The addition of NaCl resulted in concentration-dependent increases in the growth rates for both of these mutants. Thus, unlike with the *bamA101* allele, BAM activity in the E. coli Δ*waaD* strain lacking BamC or BamE was sufficient to support the growth under high membrane fluidity conditions. Elevating the membrane fluidity, for example, by raising the growth temperature to 42°C, hindered the bacterial growth of E. coli Δ*waaD* Δ*bamC* and E. coli Δ*waaD* Δ*bamE* double mutants (see Fig. S3A).

The decreased growth rates of the E. coli Δ*waaD* Δ*bamC* and E. coli Δ*waaD* Δ*bamE* double mutants under low-salt conditions (Fig. 3A and B) might have been due to compromised BAM activity. To test this, BAM activity was measured in cells by the OmpT assay, which showed decreases in E. coli Δ*waaD* Δ*bamC* and E. coli Δ*waaD* Δ*bamE* compared to that in the parent E. coli Δ*waaD* strain (Fig. 3C). Additionally, the σ^E^ periplasmic stress response that correlates with defective OMP folding was also activated by the deletion of *bamC* or *bamE* from E. coli Δ*waaD* (Fig. S3B). The overall OMP profiles were similar among all of these strains (Fig. S3C). Consistent with a reduction in BAM activity, E. coli Δ*waaD* Δ*bamC* and E. coli Δ*waaD* Δ*bamE* were 32-fold and 64-fold more sensitive to MAB1, respectively, than the parent E. coli Δ*waaD* strain (Fig. 3D). E. coli Δ*waaD* was resistant to MAB1 inhibition when grown with high NaCl (27) (Fig. 2D), and similarly, E. coli Δ*waaD* Δ*bamC* and E. coli Δ*waaD* Δ*bamE* were less sensitive to MAB1 when NaCl was added to the medium (Fig. 3E).

Because decreasing the level of the BamA target might sensitize E. coli Δ*waaD* to MAB1 inhibition, we tested if a loss of BamC or BamE influenced BamA protein levels. E. coli Δ*waaD* strains with deletions of *bamC* or *bamE* produced BamA at levels similar to that in the E. coli Δ*waaD* parent strain (Fig. 3F). Moreover, both double mutants showed similar MAB1 binding to intact E. coli cells in a fluorescence-assisted cell sorting (FACS)-based binding assay (Fig. 3G), suggesting that the increased sensitivity to MAB1 was not due to the reduced BamA target in the OM.

To determine the impact of removing BamC and BamE on the membrane, we measured fluidity in the E. coli Δ*waaD* Δ*bamC* and E. coli Δ*waaD* Δ*bamE* strains. The loss of either of the nonessential BAM lipoproteins further elevated membrane fluidity compared to that of the E. coli Δ*waaD* parental strain (Fig. 3H). These mutants also displayed increased OM permeability as measured by an increase in EtBr accumulation (Fig. S3D). In summary, deleting *bamC* or *bamE* from E. coli Δ*waaD* increased the membrane fluidity, increased the sensitivity to MAB1, and decreased BamA activity.

### E. coli Δ*bamB* and Δ*waaD* are synthetically lethal under conditions that lead to high membrane fluidity.

**FIG 4:**
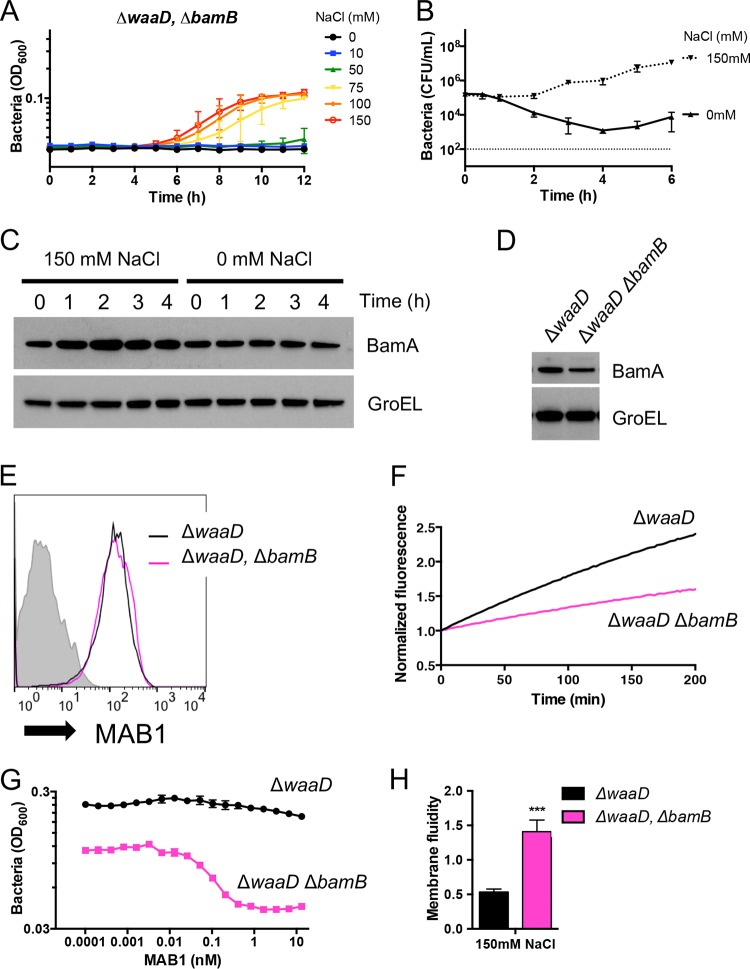
Δ*waaD* and Δ*bamB* mutations are synthetically lethal in strain grown in medium devoid of NaCl. (A) Bacterial growth curves for E. coli Δ*waaD* Δ*bamB* with increasing concentrations of supplemented NaCl are shown. E. coli Δ*waaD* Δ*bamB* loses viability when NaCl is removed. (B and C) Mid-log cultures of E. coli Δ*waaD* Δ*bamB* grown in permissive conditions were washed and resuspended in either MHB or MHB supplemented with 150 mM NaCl. CFU (B) and BamA protein level (C) were monitored over time under permissive conditions. (D and E) BamA protein levels in E. coli Δ*waaD* and E. coli Δ*waaD* Δ*bamB* were compared from mid-log cultures by Western Blot (D) and FACS (E). Representative FACS traces are shown. (F to H) OmpT substrate cleavage (F), MAB1 sensitivity (G), and membrane fluidity (H) were measured for strains grown in medium supplemented with 150 mM NaCl. Membrane fluidity data are expressed relative to E. coli Δ*waaD* grown in medium alone without NaCl. Unless noted, strains were grown in medium supplemented with 150 mM NaCl. For all plotted experiments, means and SDs from biological triplicates are shown. ***, *P < *0.001.

BamB plays a more profound role in OMP folding compared to those of BamC and BamE (6, 41). Consistent with this, we found that an E. coli Δ*waaD* Δ*bamB* double mutant, similar to the E. coli Δ*waaD bamA101* strain, was unable to grow on medium without NaCl (see Fig. S4A and B). The conditional lethality of the E. coli Δ*waaD* Δ*bamB* double mutant was suppressed by the addition of NaCl (Fig. 4A and Fig. S4A and B). Although the growth was restored when NaCl was added to the medium, the E. coli Δ*waaD* Δ*bamB* growth rate was still reduced compared to that of the parental strain (Fig. S4B). This NaCl dependence was not observed for the E. coli Δ*bamB* parental strain (Fig. S4C and D). The removal of NaCl from E. coli Δ*waaD* Δ*bamB* growth medium led to a time-dependent decrease in the number of viable bacterial cells: 4 h after washing out the NaCl, the CFU count decreased by 135-fold compared to a 7-fold increase when NaCl was present (Fig. 4B). The loss in cell viability was not due to a reduction in BamA protein levels, as BamA remained unchanged over 4 h after NaCl was removed (Fig. 4C). The intrinsic level of BamA in E. coli Δ*waaD* Δ*bamB* was reduced by ∼30% compared to that of the E. coli Δ*waaD* strain (Fig. 4D) but was still higher than the E. coli Δ*waaD bamA101* strain (Fig. 2A). Additionally, whole-cell binding by MAB1 was similar between E. coli Δ*waaD* Δ*bamB* and E. coli Δ*waaD* (Fig. 4E), indicating that the synthetic lethality was not due to dramatically reduced BamA levels. These results are consistent with a BAM defect when BamB was absent under high fluidity conditions. This hypothesis was supported by a defect observed in the OmpT activity for E. coli Δ*waaD* Δ*bamB* compared to that for the E. coli Δ*waaD* parent (Fig. 4F), the activation of the σ^E^ periplasmic stress response (Fig. S4E), and reduced OMP levels (Fig. S4F) under permissible conditions.

On the basis of the critical role for BamB under high membrane fluidity conditions and compromised BAM activity in the double mutant, we predicted that the E. coli Δ*waaD* Δ*bamB* strain would be sensitized to MAB1 activity. Under high-NaCl conditions that support growth, E. coli Δ*waaD* Δ*bamB* was completely inhibited by 2 nM MAB1, while the parent strain was resistant to the antibody under these conditions (Fig. 4G). Thus, E. coli Δ*waaD* Δ*bamB* was highly sensitized to BamA perturbation by MAB1, consistent with the observations that although BamA levels were similar, the BAM complex in this strain was less efficient. On the basis of our observations that the sensitivity to MAB1 correlates with membrane fluidity and that this appears to affect BAM activity, we predicted that membrane fluidity would be high in E. coli Δ*waaD* Δ*bamB*. Indeed, even in high-NaCl medium, which is required for the growth of this double mutant, the E. coli Δ*waaD* Δ*bamB* strain exhibited increased membrane fluidity compared to that of the parental E. coli Δ*waaD* strain (Fig. 4H). OM permeability was also elevated in this strain (Fig. S4G). Thus, our data are consistent with a defect in BAM function when BamB was absent, and this sensitized the E. coli Δ*waaD* Δ*bamB* strain to the inhibitory anti-BamA antibody MAB1, possibly through increasing membrane fluidity.

The synthetic lethality of the Δ*waaD* and Δ*bamB* mutations was overcome by growth in high NaCl, growth at low temperature, or the introduction of the LPS core oligosaccharide (Fig. 4A, 3A, and 4A to C), all conditions that, among other effects, have a commonality in that they decrease fluidity of the OM. We found previously that the loss of LpxM, which adds the sixth acyl chain to LPS (42), provided MAB1 resistance to the E. coli Δ*waaD* strain without influencing BamA levels or MAB1 binding (27). Instead, the deletion of *lpxM* reduced the excessive membrane fluidity of the E. coli Δ*waaD* strain (27). In contrast, the E. coli Δ*waaD* Δ*bamB* Δ*lpxM* triple mutant was still defective for growth in low NaCl medium and was only 4-fold less sensitive to MAB1 compared to the complete resistance gained by the loss of *lpxM* in the E. coli Δ*waaD* background (see Fig. S5A and B). Moreover, there was not a significant decrease in membrane fluidity in this E. coli Δ*waaD* Δ*bamB* Δ*lpxM* triple mutant compared to that in the E. coli Δ*waaD* Δ*bamB* strain (Fig. S5C). In this case, we suggest that the loss of LpxM was insufficient to restore rigidity to the OM to overcome the BAM defect of the E. coli Δ*waaD* Δ*bamB* strain. Consistent with this, the OmpT activity and OMP profiles of the E. coli Δ*waaD* Δ*bamB* Δ*lpxM* triple mutant were indistinguishable from those of the E. coli Δ*waaD* Δ*bamB* parent strain (Fig.S5D and S4F).

### MAB1 resistance mutations in *bamA* that do not alter antibody binding.

**FIG 5:**
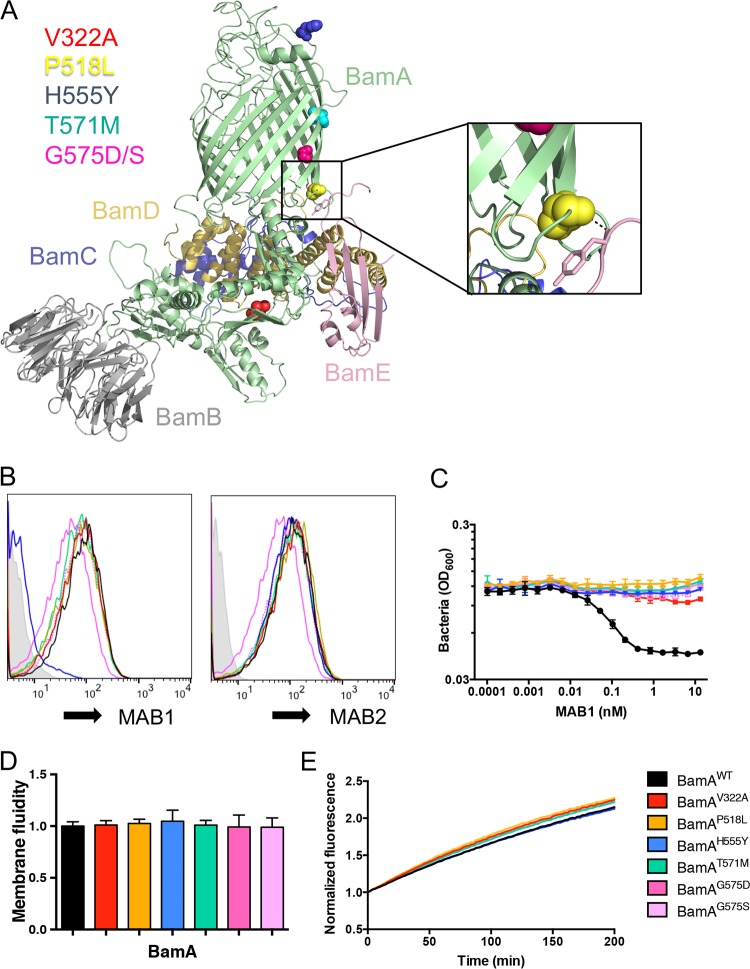
On-target MAB1 resistant *bamA* mutants. (A) Cartoon representation of BamABCDE complex from E. coli (PDB ID 5EKQ [9]). Residues V322, P518, H555, T571, and G575 are colored. Inset shows enlarged view of BamA-BamE interface. (B to E) E. coli Δ*waaD* strains producing BamA with site-directed substitutions were compared for whole-cell binding of MAB1 and MAB2 as measured by FACS (B), growth inhibition by MAB1 (C), membrane fluidity (D), and OmpT cleavage (E). Representative FACS traces are shown. For all plotted experiments, means and SDs from biological triplicates are shown.

A previous attempt to identify on-target MAB1-resistant mutants was hindered by a high frequency of off-target loss-of-function mutations in *lpxM* (27). Because the *lpxM* deletion in E. coli Δ*waaD* Δ*bamB* did not impart resistance to MAB1 (Fig. S5B), we repeated the selection for MAB1 resistance in this strain background to find novel mutations. The frequency of resistance to MAB1 was >1,000-fold lower in E. coli Δ*waaD* Δ*bamB* (∼8 × 10^−9^) than in E. coli Δ*waaD* (<1 × 10^−6^) (27). We selected 11 independent, spontaneous resistant mutants in 6 nM MAB1 under permissive growth conditions and identified *bamA* mutations in each resistant isolate. In total, six distinct point mutants were identified that resulted in changes to five BamA amino acids: V322A, P518L, H555Y, T571M, G575D, and G575S (Fig. 5A). H555Y is a known substitution located in extracellular loop 4 (L4) that disrupts MAB1 binding to BamA but not the binding of other anti-BamA antibodies such as MAB2 (27) (Fig. 5B). The five remaining BamA substitutions are new and located at distinct distal positions relative to the MAB1 binding site: T571M and G575D/S are located in the post-L4 transmembrane β-strand, P518L is located in an intracellular loop that immediately precedes the pre-L4 transmembrane β-strand and is within 4Å of BamE (9), and V322A is located within the periplasmic space in the POTRA 4 domain (Fig. 5A). Thus, MAB1 inhibition by binding to the extracellular L4 might be overcome by BamA substitutions located outside the cell, within the OM, and in the periplasmic space.

The selection of these MAB1-resistant mutants in the E. coli Δ*waaD* Δ*bamB* strain background raised the possibility that the resistance was dependent on the Δ*bamB* mutation. To eliminate this possibility, each MAB1-resistant *bamA* mutation was independently introduced into the E. coli Δ*waaD* strain; we found that all of the mutations still provided resistance to MAB1 (Fig. 5C). The H555Y substitution is known to confer resistance by preventing MAB1 binding (27), but none of the novel MAB1-resistant BamA substitutions are exposed to the cell surface (Fig. 5A). Moreover, both MAB1 and a noninhibitory anti-BamA control antibody, MAB2, bound strains containing the new BamA variants (V322A, P518L, T571M, G575D, and G575S) indistinguishably from the E. coli Δ*waaD* parent strain (Fig. 5B), indicating resistance was not imparted by preventing MAB1 binding.

We found that MAB1 activity and membrane fluidity are highly correlated. Therefore, we tested the effects of these individual *bamA* mutations on membrane fluidity. None of the E. coli Δ*waaD* strains expressing the BamA variants showed significant differences in membrane fluidity (Fig. 5D). We did observe reduced OM permeability for the G575S BamA variant (see Fig. S6A), indicating that this mutation did restore OM integrity, but the effect was presumably not sufficient to rigidify the OM, as the membrane fluidity was unchanged (Fig. 5D). Using the OmpT assay, we observed little change in BAM function using these BamA mutants (Fig. 5E). Although additional validation would be required for confirmation, E. coli Δ*waaD* strains producing BamA with P518L, V322A, T571M, and G575S substitutions showed slight, but highly reproducible, elevated OmpT activity (Fig. S6B). Overall, in addition to preventing MAB1 binding (H555Y) and decreasing membrane fluidity (by growth condition or *lpxM* deletion, for example), there is possibly a third allosteric mechanism to bypass the inhibitory effect of MAB1, as identified by these new MAB1-resistant mutants.

## DISCUSSION

Using an inhibitory anti-BamA antibody, MAB1, and mutant strains designed to disrupt, but not destroy, the BAM complex, we have described the requirements for β-barrel OMP folding in particular membrane environments. Specifically, under conditions that lead to high membrane fluidity in E. coli: (i) bacteria are less tolerant to lower levels of BamA, (ii) the nonessential BAM lipoprotein BamB is now required for growth, (iii) mutations in LPS modification can partially overcome fluidity defects (27), and (iv) mutations in the periplasmic POTRA domain and within a transmembrane β-strand of BamA provide resistance to MAB1 inhibition. In most cases, the state of the bacterial membrane environment is a predictor of the sensitivity to β-barrel OMP folding inhibition.

Adapting bacterial membranes to their environment is essential for bacterial survival. Bacteria must maintain an OM that is impenetrable to harmful compounds but flexible enough to preserve normal cell functions (1, 4). Many processes sense the environment and alter the OM composition, including membrane fluidity. For example, the growth temperature influences the proportion of saturated versus unsaturated fatty acid phospholipids, the phospholipid chain length, and LPS structure in the membrane (31, 35, 37, 38, 43, 44). Each of these affected properties influences membrane fluidity, cell physiology, and protein function. Here, we subjected E. coli to suboptimal fluidity states and found that E. coli *bamA101* Δ*waaD* and E. coli Δ*waaD* Δ*bamB* mutants were not viable. In both cases, the inability to grow was overcome in multiple ways, all of which led to decreased membrane fluidity.

The inability of E. coli *bamA101* Δ*waaD* and E. coli Δ*waaD* Δ*bamB* strains to grow highlights a potentially critical relationship between membrane fluidity and BAM function. These synthetically lethal pairs indicate that E. coli cannot simultaneously tolerate a truncated LPS, which leads to highly fluid OMs, and a decrease or loss of a BAM component under high membrane fluidity conditions. We propose that the simplest explanation for synthetic lethality in these cases is that the BAM complex alteration (i.e., decreased BamA or absence of BamB) and the OM change (i.e., absence of WaaD) both reduce the efficiency of β-barrel OMP folding, and the combined effect is detrimental to this essential cellular process. We hypothesize that this is precisely why MAB1 is active against the E. coli Δ*waaD* strain: the absence of WaaD compromises BAM activity, potentially by fluidizing the OM, and MAB1 further inhibits this process. There are other possibilities that could explain this observation. First, as both of these mutations individually activate periplasmic stress responses (27, 45, 46), it is possible that the cell cannot tolerate the combined stresses due to damage to both the lipid and the protein components of the OM. A second possibility that has not been ruled out is that the double mutants cause a backup of both OMP and LPS substrates, leading to membrane disruption. A final possibility that we cannot yet exclude is that some higher-order clustering of BAM complexes is disrupted in these synthetically lethal pairs. It was recently reported that multiple BAM complexes colocalize within precincts in the OM to facilitate the trimerization of porin OMPs (47). Interestingly, the ability of BAM complexes to form precincts requires BamB (47), which we found to be critical under high membrane fluidity conditions.

Amino acid substitutions in multiple domains of BamA can impart resistance to the inhibitory anti-BamA antibody MAB1. Altering the antibody binding site, such as in BamA H555Y, prevents MAB1 from binding to BamA on E. coli Δ*waaD* cells but does not otherwise affect the membrane or BAM activity. Here, we identified five BamA substitutions imparting MAB1 resistance (V322A, P518L, T571M, and G575D/S) that are located distally from the binding site at positions inaccessible to the antibody. In fact, even though they are resistant to inhibition, MAB1 still binds to BamA on the surfaces of these mutants. Although the molecular mechanisms of both BamA function and MAB1 activity are still unknown, the finding that mutations far removed from the MAB1 binding site can impart resistance is consistent with an allosteric model. While speculative at this point, it is possible that MAB1 binding to BamA extracellular L4 affects distal positions of BamA in other cellular compartments that are required for its β-barrel folding activity. Recent studies using single-molecule force spectroscopy showed that the POTRA domains, the composition of the membrane matrix, and extracellular loops all affect the conformation of the BamA β-barrel (48). The BamA variants described here could be important tools for deciphering such structural changes; however, it remains to be seen how these positions affect BAM structure or function.

Experiments performed on living bacterial cells under different growth conditions have enabled us to study the effects of various membrane properties on BAM activity. Optimal membrane fluidity appears to be critical for efficient BamA function, and it is possible that this facilitates some functional aspect of BamA, such as lateral gate movement, POTRA domain flexibility, or BamA stabilization upon membrane perturbation, to the extent that in extremely high fluid membranes, BamA loses its effectiveness. We propose that this is a critical consideration for future experimentation with BamA, for studying β-barrel membrane protein folding, and for screening and designing inhibitors of this process.

## MATERIALS AND METHODS

### Growth conditions.

Luria-Bertani (LB, Millers; Sigma-Aldrich L3522) and Mueller-Hinton II cation-adjusted broth (MHB; BBL 212322) were prepared according to the manufacturer’s instructions. Note that for these standard laboratory media, there is no NaCl in MHB and 171 mM NaCl in LB broth. Bacterial cultures were grown at 37°C unless otherwise stated. When appropriate, the medium was supplemented with kanamycin (50 μg/ml), carbenicillin (50 μg/ml), chloramphenicol (12.5 μg/ml), hygromycin (200 μg/ml), gentamicin (10 μg/ml), and arabinose (0.2% [vol/vol]).

### Bacterial strains and plasmids.

The bacterial strains and relevant primers are listed in Table S1 in the supplemental material. Kanamycin deletion-insertion mutants of *waaD*, *bamB*, *bamC*, and *bamE* were obtained from the Keio collection (49). Mutant alleles were created using λ Red recombination (50) and confirmed as described previously (27). All mutants were constructed and maintained on LB medium. Site-directed *bamA* mutants were created with the pBamAWT plasmid by using a QuikChange XL site-directed mutagenesis kit (Agilent) according to the manufacturer’s instructions.

### OmpT folding fluorescence assay.

The OmpT assay for monitoring BAM activity was performed as described previously with minor modifications (14, 33, 34). Bacterial strains were grown in MHB with or without 150 mM NaCl as noted in Results to early log phase and normalized to an optical density at 600 nm (OD_600_) of 0.2 in growth medium. The 50-μl solution was prepared as follows: 5 μl of bacteria was added to 45 μl fluorogenic peptide, Abz-Ala-Arg-Arg-Tyr(NO_2_)-NH_2_ (Peptide Synthesis), diluted in phosphate-buffered saline (PBS) to a final concentration of 50 μM. The mixture was immediately monitored for fluorescence on a Spectramax plate reader for 3 h, with readings every 2 min (excitation, 325 nm; emission, 430 nm). The normalized fluorescence was determined by dividing each measurement by the starting measurement.

### σ^E^ reporter assay.

Overnight cultures of bacteria possessing pGNE18 (pACAY184 plus *rpoHP3*-*lacZ*) were back-diluted to an OD_600_ of 0.01, allowed to grow to an OD_600_ of 0.2, back-diluted again to an OD_600_ of 0.01, and grown for another 2 to 3 h. At the desired time point, the cells were analyzed for β-galactosidase production by using a Beta-Glo assay (Promega) and normalized to the number of viable bacterial cells as measured using a BacTiter-Glo microbial cell viability assay (Promega). Both assays were carried out according to the manufacture’s protocols. Biological triplicates were analyzed.

### Antibody activity assay.

The screening strain was grown to log phase in MHB and, when appropriate, supplemented with NaCl as noted in Results. The cells were diluted in the same growth medium to a final OD_600_ of 0.01 in sterile round-bottom 96-well plates (Costar). The antibodies were added and bacteria were grown statically for 4 h at 37°C. The optical density of bacterial growth (OD_600_) was measured by a plate reader after shaking the plate for 25 s. CFU was measured by serially diluting the treated bacterial culture in PBS and spotting onto agar medium.

### FACS-based binding assay.

Bacterial strains were grown to log phase in MHB unless otherwise stated. Cells were harvested and resuspended to an OD_600_ of 0.5 in wash buffer (PBS supplemented with 1% bovine serum albumin [BSA]). Primary antibodies were added at 1 μg/ml and incubated at room temperature for 1 h. The cells were washed and incubated with fluorescein isothiocyanate (FITC)-conjugated secondary antibodies (1:200) for 1 h at room temperature (Life Technologies). The cells were washed and fixed in 2% paraformaldehyde (PFA) in PBS for 10 min prior to running by FACS on a FACSAria (BD) using FACSDiva software (BD).

### SDS-PAGE, Western immunoblotting, and antibodies.

Bacterial cells were grown to log phase, normalized according to the OD_600_, and pelleted. The samples were resuspended in 1× LDS sample buffer (Thermo Fisher Scientific) and boiled for 5 min prior to loading on a 4% to 12% bis-Tris SDS-PAGE gel. Proteins were transferred onto cellulose membranes using the iBlot 2 dry blotting system (Thermo Fisher Scientific). The membranes were blocked for 1 h in blocking buffer (Tris-buffered saline [TBS] containing 5% nonfat milk and 0.05% Tween 20), washed, and then incubated either overnight at 4°C or at room temperature (RT) for 1 h with the following primary Abs: mouse anti-BamA MAB2 (1 μg/ml; Genentech) and rabbit anti-GroEL (1:25,000; Enzo). Appropriate horseradish peroxidase [HRP]-linked secondary antibodies (GE Healthcare) were diluted 1:20,000 in TBS with Tween 20 (TBST) and incubated with the membrane for 1 h at RT. The blots were developed using ECL Prime Western blotting detection reagent (Amersham). Anti-BamA antibody MAB1 is a rat antibody (Genentech), the noninhibitory control anti-BamA monoclonal antibody (MAb) MAB2 is a mouse antibody (Genentech), and the antibody used for co-IP studies is an inhibitory anti-BamA MAb (MAB3) rat-variable human Fc chimera (Genentech). To make MAB3, total RNA was extracted from anti-BamA hybridoma cells (RNeasy Mini kit; Qiagen). With a SMARTer RACE cDNA amplification kit (Clontech), the RNA was first reverse transcribed and then subjected to first-strand cDNA synthesis and 5′ rapid amplification of cDNA ends (RACE) PCR of variable light and variable heavy sequences. The resulting PCR products were cloned into mammalian expression vectors containing the human kappa constant domain and human IgG1 constant domain. The recombinant MAb was obtained by transient transfections in 293 cells followed by protein A purification.

### co-IPs.

Untreated bacterial cells (150 ml) were grown to an OD_600_ of 0.5 to 0.7 in MHB. The cells were harvested by centrifugation and resuspended in 4 ml cold buffer containing 25 mM Tris (pH 8.0), 300 mM NaCl, 10% glycerol, and cOmplete EDTA-free protease inhibitor (Sigma-Aldrich) and lysed by passing through the LVI Microfluidizer homogenizer (Microfluidics). Cell debris was collected by centrifuging at 4,000 × *g* for 10 min at 4°C. The supernatant containing the whole-cell lysate was centrifuged at 40,000 × *g* for 1 h at 4°C to separate the membrane fraction from the soluble fraction. The pelleted membrane was resuspended in 1 ml of resuspension buffer (above) supplemented with 1% *n-*dodecyl β-d-maltoside (DDM) detergent. The membranes were solubilized in detergent at 4°C. The insoluble membrane fraction was removed by centrifuging the sample at 40,000 × *g* for 1 h at 4°C. The protein concentration in the soluble fraction containing the solubilized membranes was quantified with a Quick Start Bradford protein assay (Bio-Rad). A sample containing 300 μg of protein was diluted in resuspension buffer with detergent to 500 μl. Ten microliters of Dynabead protein G magnetic beads (Thermo Fisher Scientific) was added to the sample and incubated 1 h at 4°C while continuously rotating to remove nonspecific bead binding. The tubes were placed in a magnetic separation rack for 30 s. The lysate was moved to a fresh tube, and 4 μg human anti-BamA antibody (MAB7) was added for 4 h at 4°C. Subsequently, 40 μl Dynabead protein G magnetic beads was added to the mix and incubated overnight at 4°C. The beads were separated using a magnetic separation rack and washed two times in resuspension buffer with detergent. The beads were resuspended in 50 µl 1× NuPAGE LDS sample buffer supplemented with 1× NuPAGE sample reducing agent (Thermo Fisher Scientific) and heated to 95°C for 10 min. Twenty microliters of supernatant was loaded on a 12% bis-Tris SDS-PAGE gel, and proteins were stained with InstantBlue protein stain (Expedeon). For all steps at 4°C, the lysates were under continuous rotation.

### Expression and purification of the BAM complex.

An E. coli *bamABCDE* construct containing all five genes with a C-terminal 8×His tag on BamE was expressed in E. coli BL21(DE3). At an OD_600_ of 0.6, the cells were induced with 0.5 mM isopropyl-β-d-1-thiogalactopyranoside (IPTG) and harvested after 4 h at 37°C. The cells were suspended in lysis buffer containing 25 mM Tris (pH 8.0), 300 mM NaCl, 5 mM imidazole, 10% glycerol, and 1× complete protease inhibitor mixture (Roche) and lysed with a Microfluidizer at 10,000 lb/in^2^. The cell lysate was supplemented with 1% *n*-dodecyl β-d-maltoside (DDM; Anatrace) and rocked overnight at 4°C. The suspension was ultracentrifuged at 125,000 × *g* for 1 h at 4°C. The supernatant was applied to a gravity flow column (Bio-Rad) packed with 5-ml preequilibrated nickel-nitrilotriacetic acid (Ni-NTA) resin (Qiagen). The column was washed with five column volumes (CVs) of wash buffer containing 20 mM Tris-HCl (pH 8.0), 300 mM NaCl, 50 mM imidazole, 10% glycerol, and 0.03% DDM and eluted with 5 CVs of elution buffer containing 300 mM imidazole. The eluent was applied to Superdex 200 16/60 column (GE Healthcare) that had been preequilibrated with the gel filtration buffer containing 20 mM Tris (pH 8.0), 100 mM NaCl, and 1.5% *n*-octyl-β-d-glucopyranoside (OG; Anatrace).

### Cell fractionation.

Untreated bacterial cells (100 ml) were grown at 37°C with shaking to an OD_600_ of 0.8 in MHB with or without NaCl as specified. The cells were harvested by centrifugation at 4°C, resuspended in 10 ml 25 mM HEPES (pH 7.4) containing 1× cOmplete mini EDTA-free protease inhibitor cocktail (Roche), and lysed by passaging 2 times through the LVI Microfluidizer homogenizer (Microfluidics). Cell debris was collected by centrifuging at 4,000 × *g* for 10 min at 4°C. The supernatant (6 ml, whole-cell lysate) was centrifuged at 250,000 × *g* for 1 h at 4°C. The pellet containing the membranes was washed in buffer and recentrifuged. The membrane pellet was resuspended in 6 ml 25 mM HEPES (pH 7.4) with 2% sodium lauroyl-sarcosinate (Sarkosyl; Sigma) and rocked at RT for 30 min. A 1-ml sample was removed for total membrane fraction analysis. The remaining sample was centrifuged at 250,000 × *g* for 1 h at RT. The pellet containing the OM fraction was resuspended in a 10-fold smaller volume than the input volume to improve visualization. Protein samples were diluted in LDS sample buffer, heated to 95°C for 5 min, separated using a 10% bis-Tris NuPAGE gel (Invitrogen), and stained with InstantBlue protein stain (Sigma-Aldrich).

### Ethidium bromide accumulation assay.

Ethidium bromide accumulation was measured as previously described (40). Bacterial strains were grown to log phase, washed in PBS, and resuspended to an OD_600_ of 0.2. One hundred eighty microliters of cells was added to a 96-well black flat-bottom plate (Costar). Twenty microliters of ethidium bromide (100 μM) was added to the cells and PBS controls to a final concentration of 10 μM. The plate was incubated at 37°C for 2 h and then the fluorescence level was determined (excitation, 515 nm; emission, 600 nm).

### Membrane fluidity.

Membrane fluidity was measured using the Membrane Fluidity kit (Markergene/Abcam), in which a lipophilic pyrene probe is incorporated into the membranes of specific bacterial strains (27, 37). Upon membrane incorporation and spatial interaction, the monomeric pyrene probe undergoes excimer formation, dramatically shifting the emission spectrum of the pyrene probe to a longer red wavelength. By measuring the ratio of excimer (emission, 470 nm) to monomer (emission, 405 nm) fluorescence, the membrane fluidity can be quantitatively monitored. Bacterial strains were grown to log phase, washed in PBS supplemented with EDTA (1 to 4 mM depending on the strains and conditions), and labeled with labeling mix (10 μM pyrenedecanoic acid [PDA], 0.08% F-127, supplemented with EDTA in PBS) in the dark for 30 min with rocking at room temperature. The cells were washed two times in PBS and the fluorescence was measured at two wavelengths (emission, 405 nm and 470 nm; with excitation at 350 nm). To confirm membrane incorporation, the emission spectra from 380 nm to 600 nm were compared to those from unlabeled cells.

### Statistics.

All experiments examining membrane fluidity, EtBr uptake, and σ^E^ activity were analyzed via unpaired Student’s *t* tests using Prism 6.0 (GraphPad software). Bonferroni’s correction was applied to control for multiple comparisons.

## Supplementary Material

Supplemental file 1

Supplemental file 2

Supplemental file 3

Supplemental file 4

Supplemental file 5

Supplemental file 6

Supplemental file 7

Supplemental file 8
