# Supplementary figures and images for: The Escherichia coli β-Barrel Assembly Machinery Is Sensitized to Perturbations under High Membrane Fluidity
Source: J Bacteriol. 2018 Dec 7;201(1):e00517-18. doi: 10.1128/JB.00517-18 (PMC6287456; doi:10.1128/JB.00517-18)

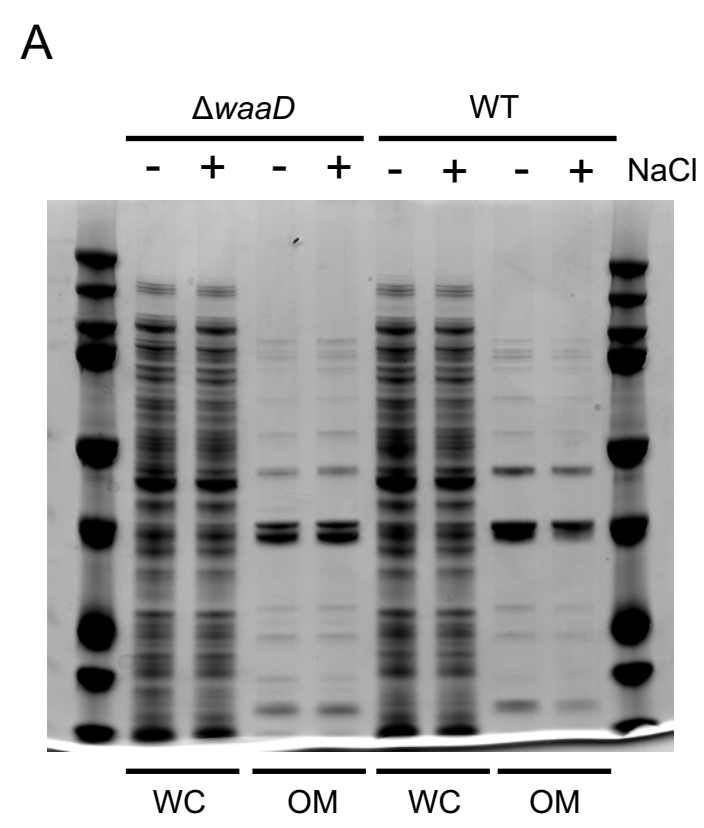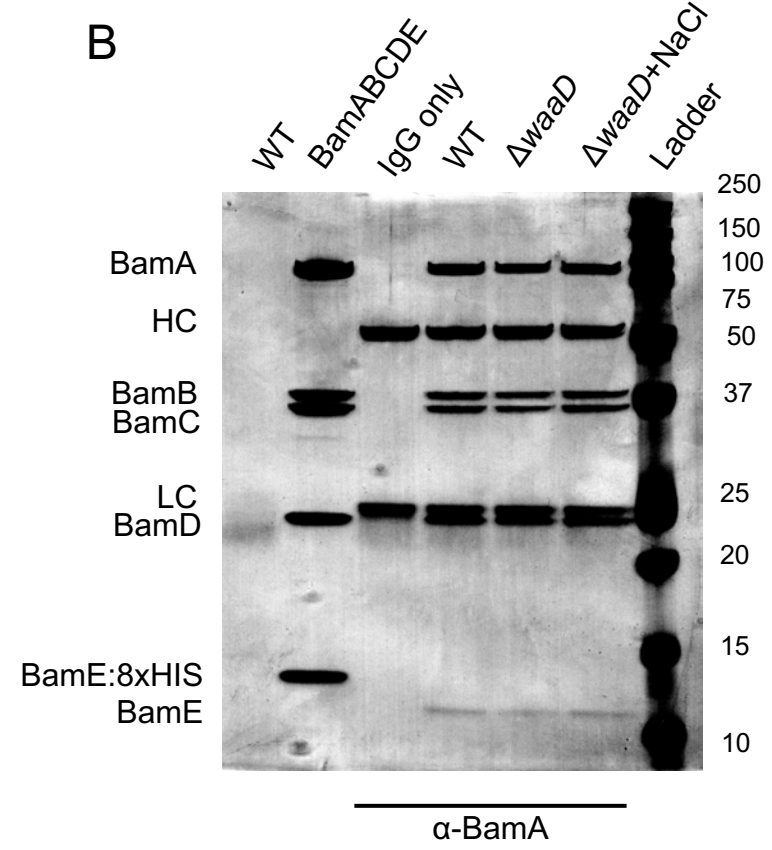

Supplement: Supplemental file 1 [file 573d4fe1e2d5647be5357ef1a900f261_JB.00517-18-s0001.pdf]

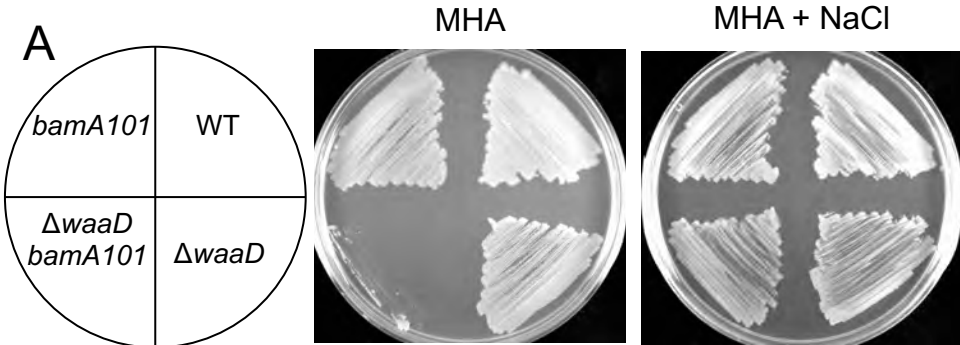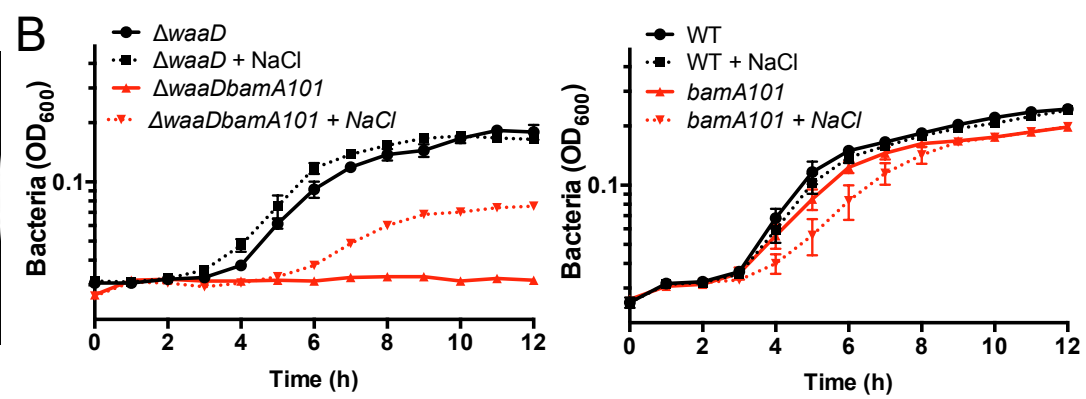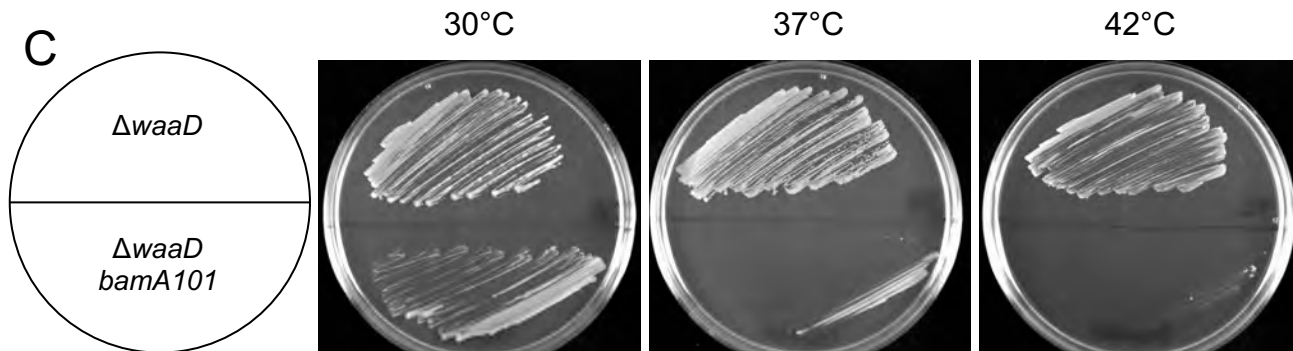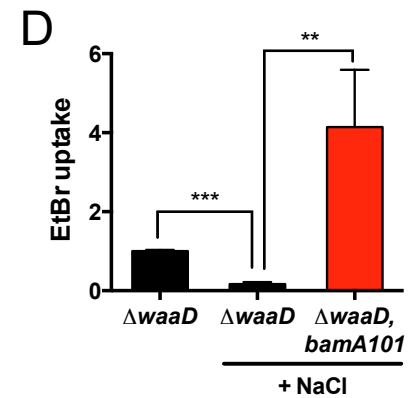

Supplement: Supplemental file 2 [file e46200aa91cab8b44ad7ca6689f0db3d_JB.00517-18-s0002.pdf]

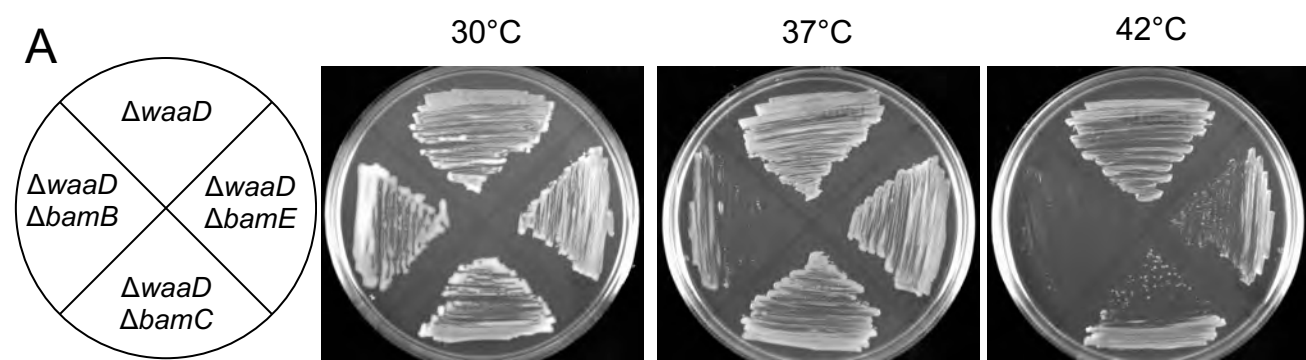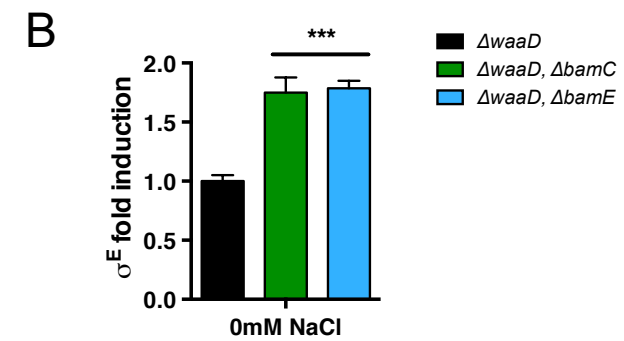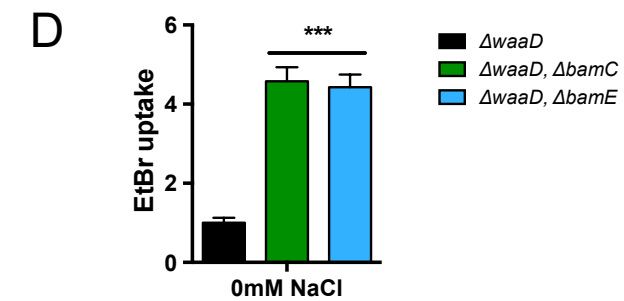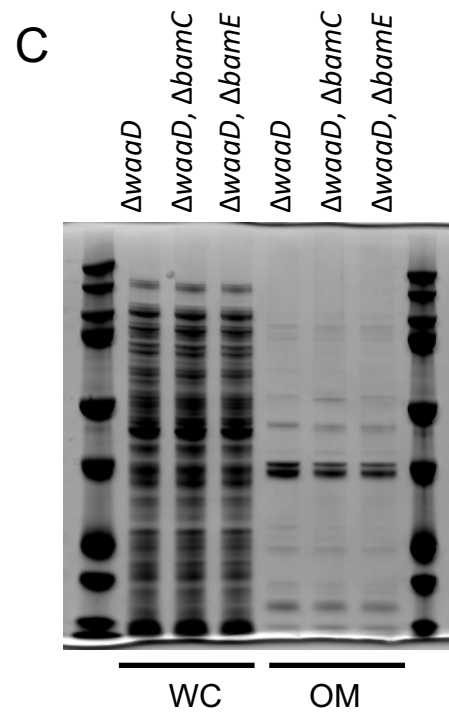

Supplement: Supplemental file 3 [file 975772c589a2ecd3cbb122f3c1cefec1_JB.00517-18-s0003.pdf]

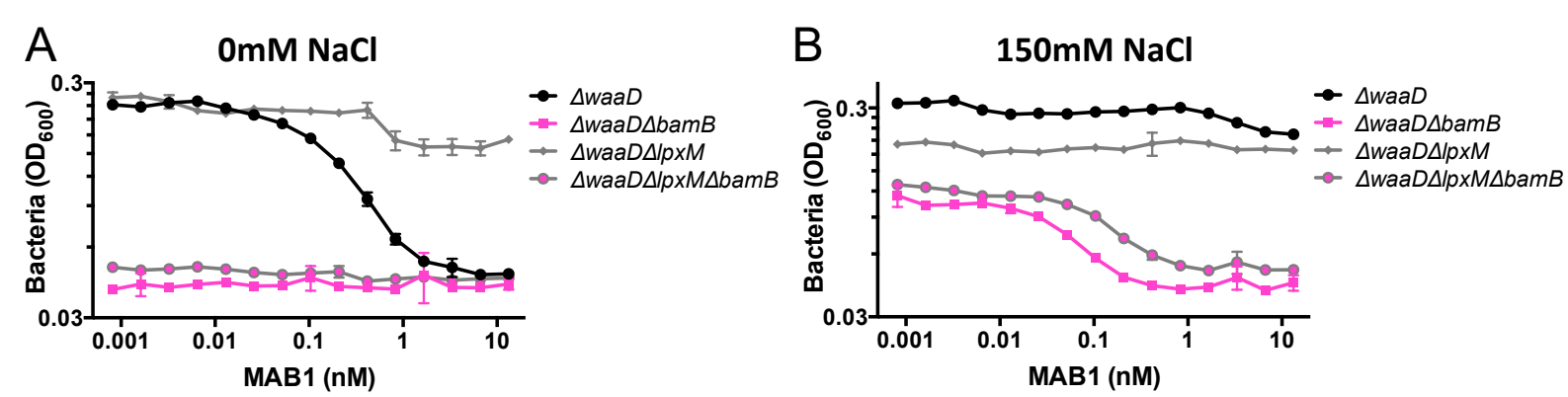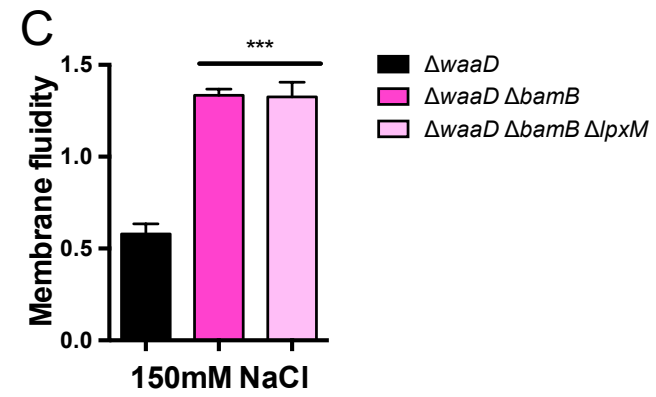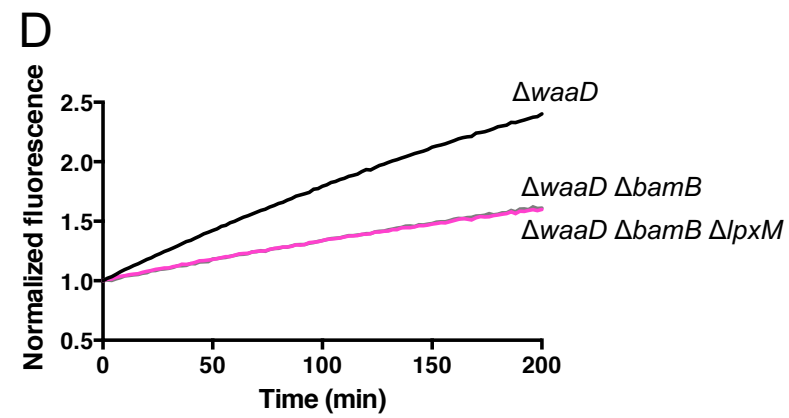

Supplement: Supplemental file 5 [file 119e74427f14a0106c523acca6a7033b_JB.00517-18-s0005.pdf]

A

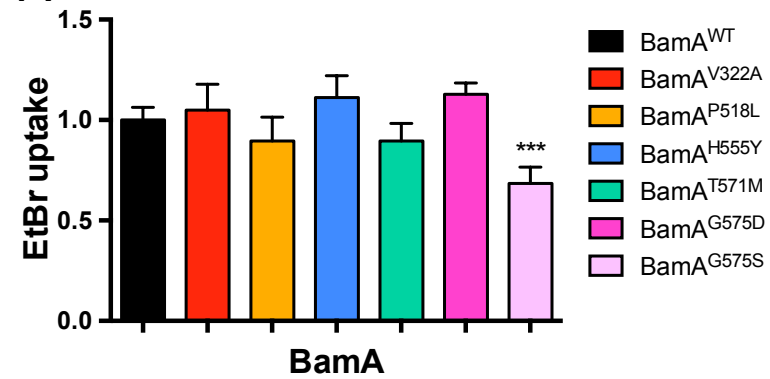

B

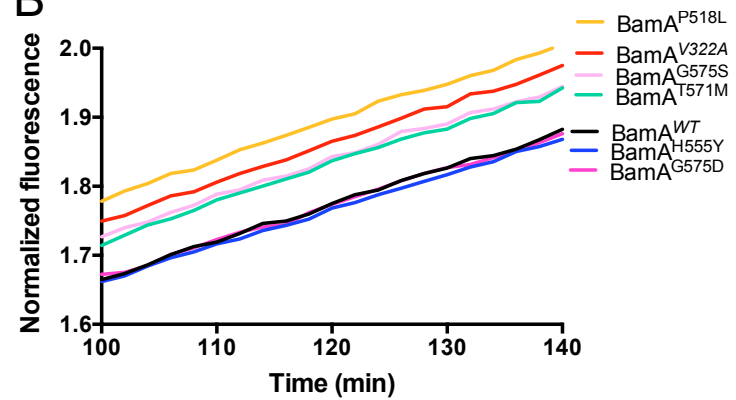

Supplement: Supplemental file 6 [file 3701b82d368f42ac9dfe51c3caf4ac2c_JB.00517-18-s0006.pdf]
