# Supplementary material for: The Escherichia coli β-Barrel Assembly Machinery Is Sensitized to Perturbations under High Membrane Fluidity
Source: J Bacteriol. 2018 Dec 7;201(1):e00517-18. doi: 10.1128/JB.00517-18 (PMC6287456; doi:10.1128/JB.00517-18)
Supplement: Supplemental file 4 [file 1a0bb0292760b47eda59aa7e2b1ff20a_JB.00517-18-s0004.pdf]

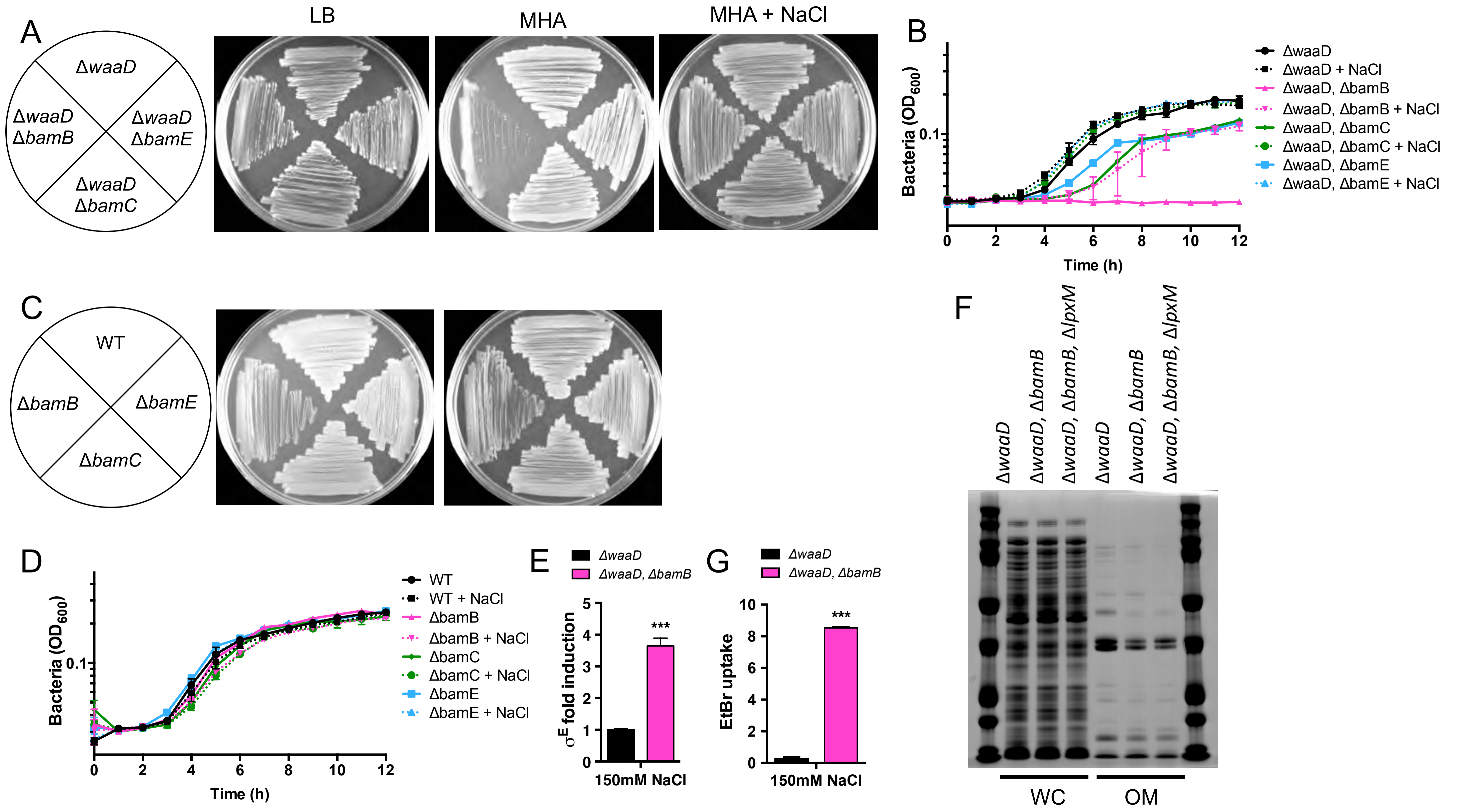

A
LB
MHA
MHA + NaCl
ΔwaaD
ΔwaaD ΔbamB
ΔwaaD ΔbamE
ΔwaaD ΔbamC
B
Bacteria (OD600)
0.1
Time (h)
0 2 4 6 8 10 12
ΔwaaD
ΔwaaD + NaCl
ΔwaaD, ΔbamB
ΔwaaD, ΔbamB + NaCl
ΔwaaD, ΔbamC
ΔwaaD, ΔbamC + NaCl
ΔwaaD, ΔbamE
ΔwaaD, ΔbamE + NaCl
C
WT
ΔbamB
ΔbamE
ΔbamC
D
Bacteria (OD600)
0.1
Time (h)
0 2 4 6 8 10 12
WT
WT + NaCl
ΔbamB
ΔbamB + NaCl
ΔbamC
ΔbamC + NaCl
ΔbamE
ΔbamE + NaCl
E
ΔwaaD
ΔwaaD, ΔbamB
σE fold induction
0 1 2 3 4 5
***
150mM NaCl
G
ΔwaaD
ΔwaaD, ΔbamB
EtBr uptake
0 2 4 6 8 10
***
150mM NaCl
F
ΔwaaD
ΔwaaD, ΔbamB
ΔwaaD, ΔbamB, ΔlpxM
ΔwaaD
ΔwaaD, ΔbamB
ΔwaaD, ΔbamB, ΔlpxM
WC
OM
