# Supplementary material for: The Escherichia coli β-Barrel Assembly Machinery Is Sensitized to Perturbations under High Membrane Fluidity
Source: J Bacteriol. 2018 Dec 7;201(1):e00517-18. doi: 10.1128/JB.00517-18 (PMC6287456; doi:10.1128/JB.00517-18)
Supplement: Supplemental file 7 [file d5c567a6156ac1a4ecdd36297ffb5193_JB.00517-18-s0007.pdf]

SUPPLEMENTARY TABLE

Table S1. Strains, plasmids, and primers used in this study

GNE ID	Strain	Plasmid	Antibiotic resistance	Origin
GNE 49	BW25113 Δ*waaD::Kan*		Kan-R	Keio collection
GNE 115	BW25113			
GNE 4741	BW25113 Δ*bamB::Kan*		Kan-R	Keio collection
GNE 4742	BW25113 Δ*bamC::Kan*		Kan-R	Keio collection
GNE 4743	BW25113 Δ*bamE::Kan*		Kan-R	Keio collection
GNE 142	BW25113 *bamA101::Kan*		Kan-R	Aoki 2008
GNE 4062	BW25113 Δ*bamB::Kan, ΔwaaD::Gent*		Kan-R, Gent-R	in-house
GNE 4063	BW25113 Δ*bamC::Kan, ΔwaaD::Gent*		Kan-R, Gent-R	in-house
GNE 4064	BW25113 Δ*bamE::Kan, ΔwaaD::Gent*		Kan-R, Gent-R	in-house
GNE 4286	BW25113 *bamA101::Kan, ΔwaaD::Cm*		Kan-R, Cm-R	in-house
GNE 491	BW25113 Δ*waaD, ΔlpxM*			Storek 2018
GNE 4089	BW25113 Δ*waaD, ΔlpxM, ΔbamB::Kan*		Kan-R	in-house
GNE 1101	Δ*bamA*::P_{BAD}-*bamA, ΔwaaD::Cm*		Carb-R, Kan-R, Cm-R	Storek 2018
GNE 3944	Δ*bamA*::P_{BAD}-*bamA, ΔwaaD::Cm*	p*bamA WT*	Carb-R, Kan-R, Cm-R, Gent-R	Storek 2018
GNE 4153	Δ*bamA*::P_{BAD}-*bamA, ΔwaaD::Cm*	p*bamA H555Y*	Carb-R, Kan-R, Cm-R, Gent-R	Storek 2018
GNE 4832	Δ*bamA*::P_{BAD}-*bamA, ΔwaaD::Cm*	p*bamA V322A*	Carb-R, Kan-R, Cm-R, Gent-R	in-house
GNE 4799	Δ*bamA*::P_{BAD}-*bamA, ΔwaaD::Cm*	p*bamA P518L*	Carb-R, Kan-R, Cm-R, Gent-R	in-house
GNE 4801	Δ*bamA*::P_{BAD}-*bamA, ΔwaaD::Cm*	p*bamA T571M*	Carb-R, Kan-R, Cm-R, Gent-R	in-house
GNE 4800	Δ*bamA*::P_{BAD}-*bamA, ΔwaaD::Cm*	p*bamA G575S*	Carb-R, Kan-R, Cm-R, Gent-R	in-house
GNE 4833	Δ*bamA*::P_{BAD}-*bamA, ΔwaaD::Cm*	p*bamA G575D*	Carb-R, Kan-R, Cm-R, Gent-R	in-house

plasmids	Description	Antibiotic resitance	Origin
p*bamAWT*	pBla_short bamA (*E. coli*)	Carb-R, Gent-R	Storek 2018
pKD4	lambda-red recombination	Kan-R	Addgene
pSIM18	Expresses Lambda Red recombinase	Hygro-R	Chan, W. et al. 2007
pCP20	ts FLP expression plasmid	Carb-R, Cm-R	Coli Genetic Stock Center
prpoHP3-lacZ	pACYC184 rpoHP3:lacZ	Tet-R, Cm-R	Storek 2018

Primers	Sequence
Knock-outs	
waaD KO F	GTCTGAGATTGTCTCTGACTCCATAATTCGAAGGTTACAGTTATGATCATCgtgtaggctggagctgcttc
waaD KO R	CCCAAGACGGGCCGATCACCAGTATTTTCATGCAGAGCTCTTATGCGTCGCGcatatgaatatcctccttagttcctattc
bamB KO F	atgCAATTGCGTAAATTACTGCTGCCAGGACTGCTTTCCGTTACCCTTTTAAGCGgtgtaggctggagctgcttc
bamB KO R	ttaACGTGTAATAGAGTACACGGTTCCGTCTTTTGCCTGGATCAGCAGTTTGCCcatatgaatatcctccttagttcctattc
pBla_BamA point mutants	
BamA QC P518L F	CGTTGGGCTTCCtGATTAACGAATATAAC
BamA QC P518L R	GTTATATTCGTTAATCaGGAAGCCCAACG
BamA QC G575S F	GACTTCACGTTCAACTATaGTTGGACCTATAACAAGC
BamA QC G575S R	GCTTGTTATAGGTCCAACtATAGTTGAACGTGAAGTC
BamA QC T571M F	CGGACGACTTCAtGTTCAACTATGG
BamA QC T571M R	CCATAGTTGAACaTGAAGTCGTCCG
BamA QC G575D F	CGACTTCACGTTCAACTATGaTTGGACCTATAACAAGC
BamA QC G575D R	GCTTGTTATAGGTCCAAtCATAGTTGAACGTGAAGTCG
BamA QC V322A F	CCTATCCGCGCGcACAGTCGATGCCCG
BamA QC V322A R	CGGGCATCGACTGTgCGCGCGGATAGG
