# Supplementary material for: The Escherichia coli β-Barrel Assembly Machinery Is Sensitized to Perturbations under High Membrane Fluidity
Source: J Bacteriol. 2018 Dec 7;201(1):e00517-18. doi: 10.1128/JB.00517-18 (PMC6287456; doi:10.1128/JB.00517-18)
Supplement: Supplemental file 8 [file d187fef6d1b9196a4c726677aafe8b1f_JB.00517-18-so008.pdf]

SUPPLEMENTARY FIGURE LEGENDS

Figure S1. OMP profiles and BAM complexes for WT and Δ*waaD* *E. coli*. (A) Coomassie-strained SDS-PAGE of whole cell lysates (WC) and outer membranes (OM) isolated from bacteria grown with or without 150 mM NaCl. The OMs were concentrated 10-fold to improve visualization. (B) Figure 1B enhanced for contrast to visualize BamE (12kDa) in the co-IP experiments described in Figure 1.

Figure S2. *E. coli* Δ*waaD bamA*101 is synthetically lethal in low NaCl growth conditions. (A) Colony growth of *E. coli* wild-type (WT), *E. coli bamA*101, *E. coli* Δ*waaD*, and *E. coli* Δ*waaD bamA*101 on media alone (left) or media supplemented with 150 mM NaCl (right). (B) Growth curves of (left) *E. coli* Δ*waaD* and *E. coli bamA*101 Δ*waaD* or (right) *E. coli* wild-type (WT) and *E. coli bamA*101 grown in the presence or absence of 150 mM NaCl. (C) Colony growth of *E. coli* Δ*waaD* and *E. coli* Δ*waaD bamA*101 on agar media grown at 30°C, 37°C and 42°C. (D) Ethidium bromide uptake for strains grown in media supplement with 150 mM NaCl. Means and SDs of biological triplicates are shown. **$p < 0.01$, ***$p < 0.001$.

Figure S3. BamC and BamE improve BAM activity and membrane integrity. (A) Colony growth of *E. coli* Δ*waaD, E. coli* Δ*waaD* Δ*bamB, E. coli* Δ*waaD* Δ*bamC* and *E. coli* Δ*waaD* Δ*bamE* on agar media grown at 30°C, 37°C and 42°C. (B) σ^E expression, (C) OMP profiles, and (D) EtBr accumulation were measured for strains grown in media alone. Coomassie-strained SDS-PAGE of whole cell lysates (WC) and outer membranes (OM) isolated from bacteria grown

without 150 mM NaCl. The OMs were concentrated 10-fold to improve visualization. Means and SDs of biological triplicates are shown. ***$p < 0.001$.

Figure S4. Δ*bamB* and Δ*waaD* are synthetically lethal. Colony growth of Δ*bamB*, Δ*bamC* and Δ*bamE* mutants on indicated media in the parent background of (A) *E. coli* Δ*waaD* or (C) *E. coli* wild-type (WT). Growth curves of *E. coli* Δ*bamB*, Δ*bamC* and Δ*bamE* mutants grown in the presence or absence of 150 mM NaCl in (B) *E. coli* Δ*waaD* or (D) wild-type *E. coli*. (E) σ^E expression, (F) OMP profiles, and (G) EtBr accumulation were measured for strains grown in media supplemented with 150 mM NaCl. The OMs were concentrated 10-fold to improve visualization compared to whole cells (WC). Means and SDs of biological triplicates are shown. ***$p < 0.001$.

Figure S5. *lpxM* deletion does not restore complete MAB1 resistance to *E. coli* Δ*waaD* Δ*bamB*. Bacterial growth inhibition by MAB1 measured by bacterial cell density (OD_{600}) in (A) media alone or (B) media supplemented with 150 mM NaCl. (C) Membrane fluidity and (D) OmpT cleavage assay were compared for strains grown with 150 mM NaCl. Membrane fluidity was normalized to *E. coli* Δ*waaD* grown without NaCl. Means and SDs of biological triplicates are shown. ***$p < 0.001$.

Figure S6. On-target MAB1 resistant *bamA* mutants. (A) EtBr accumulation was compared for strains grown in media alone. (B) OmpT substrate assay from Figure 5E zoomed to enhance visual comparisons. Means and SDs of biological triplicates are shown. ***$p < 0.001$.
